# From “Husky” to “Bulldog”– behavioural correlates between castration and breed groups in the domestic dog (*Canis lupus familiaris*)

**DOI:** 10.1186/s12917-024-04097-6

**Published:** 2024-06-03

**Authors:** C.A. Kolkmeyer, J. Baum, N. Warlich-Zach, U. Gansloßer

**Affiliations:** 1 Department of Biology, University of Vechta, driverstrasee 22, 49377 Vechta, Germany; 2 Institut für Zoologie & Evolutionsforschung mit Phyletischem Museum, Ernst-Haeckel-Haus und Biologiedidaktik, Erbertstr.1, Jena 07743 Thuringia, Germany

**Keywords:** Neutering, Castration, Breed, Husky, Bulldog, Behaviour

## Abstract

Neutering dogs is a widespread method and is carried out for various behavioural and husbandry reasons. This study’s main objective is to investigate the behavioural correlations between neutering and the breed of male dogs. In order to possibly find breed-dependent differences in the behaviour of intact and castrated dogs, a differentiation between two clades - the “Huskies“(chow chow, shar pei, akita/shiba inu, alaskan malamute, siberian/alaskan husky) and the “Bulldogs” (german boxer, english/french bulldog, old english mastiff, boston terrier, english bull terrier, staffordshire bull terrier, american staffordshire terrier), based on Parker et al. [1], was made.

Using an online questionnaire,, 31 neutered and 37 intact male dogs from the clade “Huskies“ and 30 neutered and 38 intact male dogs from the clade “Bulldogs”, participated in the study (N = 136).The survey included detailed questions on the dogs’ personality and any associated issues as well as a behavioural anamnesis. Further questions relating to four of the “big five” personality dimensions based on the “Budapest questionnaire” by Turcsán et al. from 2011 [2] were also added.

The results show, that neutered males from both breed clades more frequently displayed aggression toward humans than intact males (multinomial logistic regression, *p* = 0.002). When it came to aggression towards other dogs, it was the “Huskies” that differed significantly from the “Bulldogs“(multinomial logistic regression, *p* = 0.04) with being more aggressive. There were also significant differences in stress-related behaviour depending on castration status and breed (multinomial logistic regression, *p* < 0.001; Cramer’s V = 0.33) and only the castration status had an impact on the significance (multinomial logistic regression, *p* < 0.001). The analysis also revealed significance for stress-indicating behaviour with dependence on neutering status (multinomial logistic regression, *p* < 0.001) and showed that stress as well as uncertainty are significantly more common in neutered dogs depending on breed and neutering status (multinomial logistic regression, *p* < 0.001; Cramer’s V = 0.42), in that only neutered “Bulldogs“ were stressed, but more “Huskies“ overall.

According to the Budapest questionnaire data, the “Bulldog” clade had considerably greater extraversion scores overall (ordinal regression, *p* < 0.001) than the “Huskies“.

Our findings highlight the risks and potential negative effects of neutering. Gonadectomy in no way substitutes for the dog receiving the necessary socialization, training, or bonding. Although in some circumstances it might have a favourable impact on the dog’s behaviour, it should not be seen as a panacea for unwanted behaviour. Given that not all behaviours are influenced by sex hormones, every castration decision must be weighed up individually.

**Supplementary Information:**

The online version contains supplementary material available at 10.1186/s12917-024-04097-6.

## Introduction

Neutering dogs is a routine procedure nowadays. While some dog owners opt for neutering for medical aspects, others hope for positive effects on certain behavioural problems. Nevertheless, it can never be predicted with certainty how a dog will change after neutering [3].

Dog owners decide to get their dogs neutered for a variety of reasons. They often expect health or behavioural benefits such as the prevention of testicular and prostate disease in male dogs or the reduction of different behaviours such as roaming or marking [3, 4]. However, studies in recent years have shown that the hoped-for benefits ultimately do not come into effect as previously assumed and that neutering can also have negative health effects such as joint disease [4, 5] or immune deficits [6].

Kaufmann et al. [7] indicate that neutering can cause unwanted social behaviour of dogs. Based on video analyses and questionnaires it could be found that castrated males were significantly more aggressive, more panicky and less sociable compared to the intact males (personality scores based on Turcsán et al. [2]). Further studies by Lorenz et al. [8, 9] came to similar results in the behaviour of female dogs. In addition, current research suggests that problems associated with neutering may depend on the age or breed of the dog [10, 11].

However, there are also studies in which no connection between behavioural problems and castration was found.

According to Palestrini et al., neutered male dogs showed less riding up, pulled less on the leash and showed less owner-directed aggression, according to their owners [12].

Serpell and Hsu, in turn, concluded that castrated Sheepdogs are more trainable than the intact ones [13].

In addition to the studies in which a negative influence of castration on aggressive behaviour was observed [14, 15], there are also studies in which aggressive behaviour in female dogs appears to be independent of castration [16], whereas sensitivity to noise or a more intense fear response was higher in the castrated dogs [16]. Conversely, positive effects of castration on aggression have also been reported [17, 18].

In Germany, the castration of dogs is one of the most frequently performed surgical procedures in small animal practices [19]. For some breeds, there is also a dog regulation act, which, depending on the federal state, prescribes the castration/sterilization of dog breeds classified as potentially dangerous. This mainly affects the dog breeds Staffordshire Bull Terrier, Bulldog and Mastiff (regulations for dangerous dogs 2018 [20].

One of the main reasons for castration is the avoidance of aggressiveness, but the topic of aggression in dogs is extremely multifaceted. There is more than one type of aggression and it is not limited to certain breeds [21–23]. Rather it is a combination of different influencing factors that can lead to aggression. Thus, it is very important to distinguish between different forms of aggression [24].

One hormone that plays a major role in the decision regarding castration is cortisol. As a component of the stress system, cortisol is modulated by some messenger substances (e.g. serotonin and oxytocin) and also by sex hormones. Serotonin, oxytocin and sex hormones (such as testosterone and estrogen) compete with cortisol. Their mode of action is therefore stress- or cortisol-reducing and fear-relieving [25–27]. Related to this, Salavati et al. [28] found increased cortisol and low serotonin levels in neutered dogs. These findings are supported by recent behavioural studies in which neutered males were also more fearful, panicky and fear-aggressive than their intact conspecifics [3, 7, 10, 29].

A similar form of fear-related aggression that is not sexual in nature is defensiveaggression [30, 31]. This aggression is one of the common and often misinterpreted type of aggression. It is influenced by stress hormones and occurs primarily in stressful situations [32, 33].

Another example would be food-related aggression: this would also be worsened by neutering, as cortisol is primarily involved [34].

In addition to many reasons for neutering, which have been invalidated by current studies, many dog owners also cite reasons of convenience as a reason for neutering, without considering the possible consequences for the dog [35, 36]. However, such a castration for a more comfortable life with the dog is against the German animal welfare act (§ 6, German Animal Welfare Act [19]), .

Regarding differences in the social behaviour of dogs, the question of breed-dependent behaviour naturally arises. However, the questionnaire study by Kolkmeyer et al. [10] showed that the dog breed may not have as great an effect as often assumed. They compared neutered (*n* = 112) and intact males (*n* = 130) from four breed categories of “Shepherds, Retrievers, Terriers, Hunting Dogs”, (sensu Parker et al. [1]) with each other. The neutered males, regardless of breed, showed less extroverted behaviour (towards dogs) than intact dogs. Starling et al. [37] also found a significant effect of breed on the personality category “boldness.” In this, retrievers were bolder overall than hunting dog breeds and the loose-eyed herding breeds were bolder than heading and cattle-herding breeds.

In 2017, Parker et al. published a genetic study that took a new perspective at the classification of dog breeds under consideration of migration, geographical separation and remixing [1]. This genome analysis ultimately resulted in the modern breed classification, which is referred to as clades. The researchers were able to identify a total of 23 clades. A selection of dog breeds is assigned to each clade.

The focus of this study is to investigate the behavioural correlates between neutering and two specific breed clades (sensu Parker et al. [1]) in the domestic dog. For this purpose, a differentiation is made between two clades, namely the clade of “Huskies” (Chow Chow, Shar Pei, Akita/Shiba Inu, Alaskan Malamute, Siberian/Alaskan Husky; a clade referred by Parker et al. [1] as clade A (“Akita”)) and the clade of “Bulldogs” (German Boxer, English/French Bulldog, Old English Mastiff, Boston Terrier, English Bull Terrier, Staffordshire Bull Terrier, American Staffordshire Terrier; a clade referred by Parker et al. [1] as clade W (“English Mastiff”)), in order to possibly find breed-dependent differences in the behaviour of intact and castrated dogs. Because of an unequal representation of the different breeds in our study is also the reason for us to rename the clades, different from the naming in Parker et al. [1].

We have selected these two clades, since we have already involved some other breed clades in another study [10] and wanted to cover the 360° of the cladogram of Parker et al. [1] as far as possible. In addition, we are also interested in the original Nordic dog types because of their more wolf-like annual rhythm compared to a clade as far away from it as possible [38]. Details on the breed portraits concerning the character traits of the participating dog breeds can be found on the website of the Kennel Club [39].

Due to the complex effects of castration and its numerous influencing factors, the following hypotheses are put forward based on publications by Kaufmann et al. [7], Lorenz et al. [8, 9] and Kolkmeyer et al. [10]. (H1) Neutered males are (breed-dependently) more often aggressive than intact males. (H2) Neutered males show (breed-dependently) more stress-indicating behaviour (stress/insecurity, fear of noises, problems with dogs, stress towards people, stress for other reasons, panting, licking/scratching and stereotypies according to Handelman [40]) than intact males. (H3) There is a difference of scores for the four personality traits of calmness, trainability, sociability and extraversion (sensu Turcsán et al. [2])., between the neutered and intact males (depending on their breed).

**Table 1:** Distribution of the participating dogs from clade 7 and clade 9 (sensu Parker et al. [1]) based on their respective breed

Clade “Husky”	Number of dogs			Clade “Bulldog”	Number of dogs		
	Neutered	Intact	Total		Neutered	Intact	Total
Chow-Chow	1	1	**2**	German Boxer	2	7	**9**
Chinese Shar Pei	9	16	**25**	English Bulldog	12	23	**35**
Akita Inu	5	4	**9**	French Bulldog	8	1	**9**
Shiba Inu	2	2	**4**	Old English Mastiff	1		**1**
Alaskan Malamute	3	6	**9**	Boston Terrier		2	**2**
Siberian Husky	9	8	**17**	English Bull Terrier	1		**1**
Alaskan Husky	1		**1**	Staffordshire Bull Terrier	2	1	**3**
				American Staffordshire Terrier	4	4	**8**
Chow-Chow-Husky-Mix	1		**1**				
Total	**31**	**37**	**68**	**total**	**30**	**38**	**68**

## Materials and methods

### Subjects

Data was collected between 2020 and 2023 using two online questionnaires (umfrageonline.de & survio.de). To recruit participants, notices were posted in (pet supply) shops and flyers were distributed to friends, veterinarians and associations as well as to participants of social networks in German speaking countries.

### Questionnaires

A behavioural anamnesis was recorded in the survey with specific questions about the dogs’ personality and possible related problems (the questionnaire can be found in the supplementary data). The questionnaires on behavioural anamnesis originate from our working group, the Mammalia AG, and have already been used in numerous studies [7, 9, 10, 41] and have since functioned as a valid test instrument.

An additional questionnaire based on Turcsán et al. ( [2], “Budapest questionnaire”) was also included. This questionnaire was developed by the aforementioned working group and offers a possibility to measure the calmness, trainability (which corresponds more to the big-five-trait openness), sociability and extraversion (termed boldness, but renamed here due to the term “boldness” having a different, more comprehensive meaning of behavioural or evolutionary ecology [42, 43]. Dogs with a higher score within the personality indicate a stronger expression of the respective trait (for detailed information see Table 2).

**Table 2:** The variables of the anamnesis Questionnaire with their belonging to the “stress”, “nervousness” or “aggression” category and the four personality traits of the Budapest Questionnaire based on Turcsán et al. [2]

Stress	Nervousness	Aggression	Budapest Questionnaire
Uncertainty	Licking/scratching	Aggression in general	Emotional stability
Noises	Seems absent	On the walk	Trainability
Dogs	Never getting tired	Towards dogs	Extraversion
Humans	Restlessness	Humans	Sociability with dogs
Panting	Unreasonably nervous	Humans of the same household	
Stereotypic behaviour			
Other			

### Data analyses

The characteristic values of the anamnesis questionnaire were formulated as dichotomous variables with the corresponding values true and false. Within the Budapest questionnaire (by Turcsán et al. [2]) there is a three-point scale, in which participants could select to what extend statement applies to their dog. In general, the MICROSOFT® EXCEL® (2016) programme was used to collect and analyse the data and to create the diagrams. The statistical analysis of the data was carried out with the help of the statistical software IBM® SPSS® Statistics (2022).

Due to the small sample size and the nominal/ordinal data level there are non parametric conditions. Multinomial logistic regressions were performed to test the influence of different variables (neuter status and breed) simultaneously. For the analysis of the Budapest questionnaire an ordinal regression analysis was performed [44].

To calculate the magnitude of the relationship between the variables, effect size was determined. For the combined effects (castration status and breed) Cramer’s V [45, 46] was calculated. The ranges for interpreting the indices of this effect size according to Funder and Ozer [45] are:

*r* < 0.05 – tiny.

0.05 < = *r* < 0.1 - very small.

0.1 < = *r* < 0.2 - small.

0.2 < = *r* < 0.3 - medium.

0.3 < = *r* < 0.4 - large.

r > = 0.4 - very large.

For single effects (either castration status or breed), the odds ratio [47] was applied with the following ranges according to Chen et al. [48]:

Exp(B) < 1.68 - very small.

1.68 < = Exp(B) < 3.47 - small.

3.47 < = Exp(B) < 6.71 - medium.

Exp(B) > = 6.71 - large.

## Results

A total of 31 neutered and 37 intact males of the “Huskies“ clade and 30 neutered and 38 intact of the “Bulldogs” clade participated in the study (N = 136). There are some breeds of the clades that are represented more frequently and others being not represented at all (see Table 1).

Our results show that the dogs differ in certain characteristics, either in dependence on breed, in dependence on neutering status, or in relation to both variables.

**Fig. 1:**
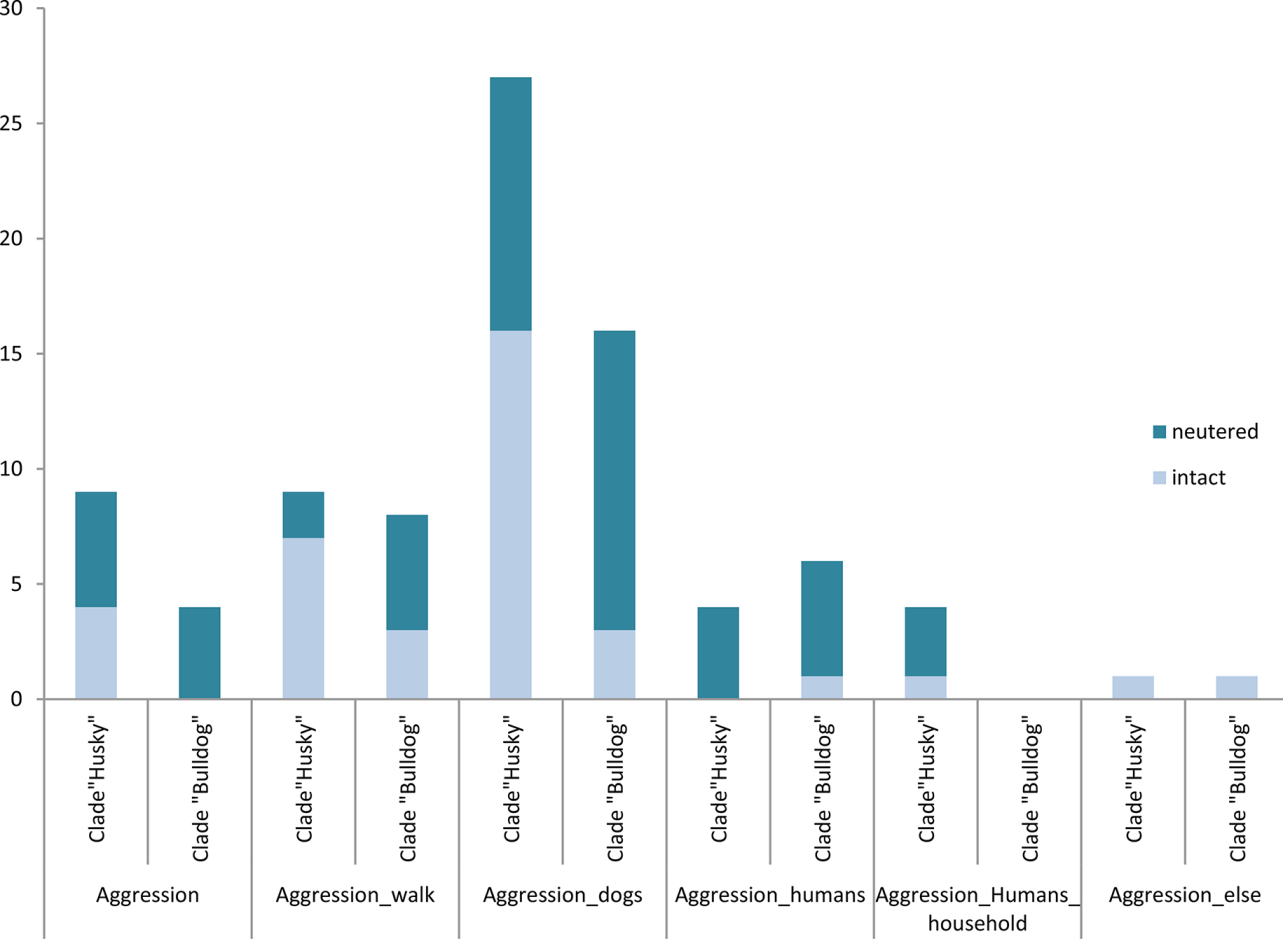
Aggression: Comparison of neutered (*n* = 31) and intact males (*n* = 37) of the clade “Huskies” with the neutered (*n* = 30) and intact males (*n* = 38) of the clade “Bulldogs” regarding aggressive behaviour depending on their breed and neutering status. There are significant differences concerning aggression towards dogs (multinomial regression analysis: final significance *p* = 0.03; for breed *p* = 0.04), aggression towards humans (multinomial regression analysis: final significance *p* = 0.006; for neuter status: *p* = 0.002), aggression towards people of the same household (multinomial regression analysis: final significance *p* = 0.03, for breed *p* = 0.02)

When comparing aggressive behaviour, there were especially large differences in general aggression (multinomial regression, *p* = 0.06) and aggression towards other dogs (multinomial regression, *p* = 0.03, Cramer’s V = 0.23) as well as towards humans (multinomial regression, *p* = 0.006, Cramer’s V = 0.28) between the neutered and intact “Huskies” and “Bulldogs” (see Fig. 1).

In the trait “aggression towards humans”, the proportion of neuters showing these behaviours was higher than that of intact dogs (multinomial regression, *p* = 0.002, OD = 0.08).

While there are more intact than neutered males with aggressive behaviour in general and aggression on the walk within the “Husky” clade, there are more neutered males showing these traits within the “Bulldog” clade.

Aggression towards people of the same household only occurs in “Huskies” (multinomial regression, *p* = 0.02 for the variable breed).

**Fig. 2:**
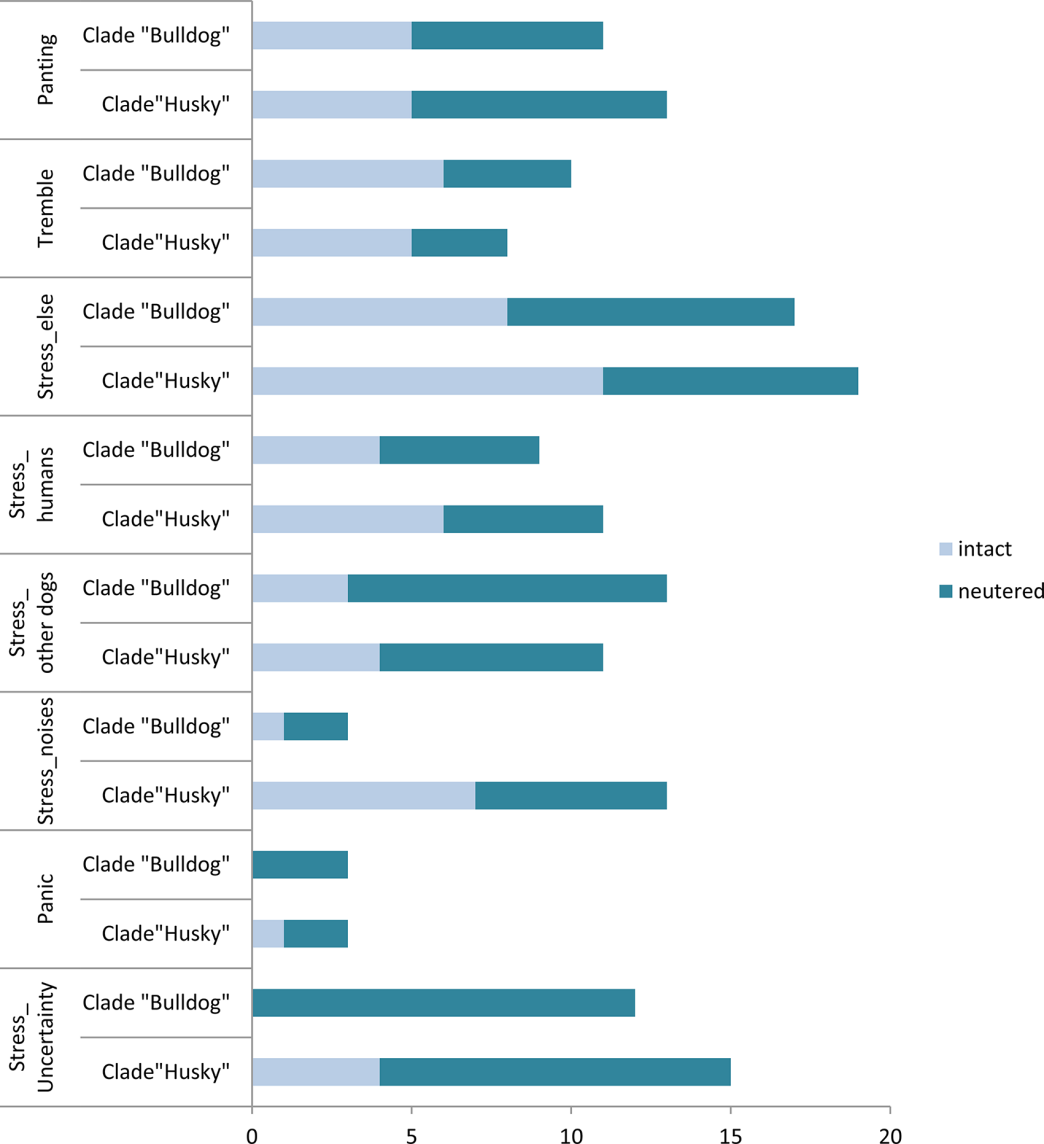
Stress-indicating behaviour: Comparison of neutered (*n* = 31) and intact males (*n* = 37) of the clade “Huskies” with the neutered (*n* = 30) and intact males (*n* = 38) of the “Bulldog” clade with regard to stress-indicating behaviour depending on their breed and neutering status. There is a significance for stress due to dogs and noises (multinomial regression analysis: *p* = 0.02; for neuter status (stress with dogs): *p* = 0.004; for breed (stress with noises): *p* = 0.006), stress and uncertainty depending on both breed and neutering status (multinomial regression analysis: final significance *p* < 0.001) and also a significance for the dependence only on neutering status (multinomial regression analysis: *p* < 0.001)

In stress-indicating behaviour, there are also differences between neutered and intact males (Fig. 2). Again, characteristics such as stress and uncertainty as well as stress in relation to other dogs or noises stand out, which occur more frequently in the neutered than in the intact dogs. Stress and uncertainty are significantly more frequent in neutered dogs depending on breed and neutering status (multinomial regression analysis: final significance *p* < 0.001) with a very large effect size (Cramer’s V = 0.42). Depending only on the castration status, the significance was *p* < 0.001 (multinomial regression analysis). It is noticeable that only neutered and not intact “Bulldogs” show stress and insecurity.

Stress due to dogs or noises was significant, too, each with equal p-values (multinomial regression analysis, *p* = 0.02) and the analysis showed significance for the dependence on neutering status for stress in relation to other dogs (multinomial regression analysis: *p* = 0.004). The high values for stress in case of noises within the “Huskies” are striking (multinomial regression analysis: *p* = 0.006) and have a large odds ratio (OD = 5.11). Additionally, there was a significant difference within panic behaviour due to the castration status with more neutered dogs being panicky (multinomial regression analysis: *p* = 0.05).

**Fig. 3:**
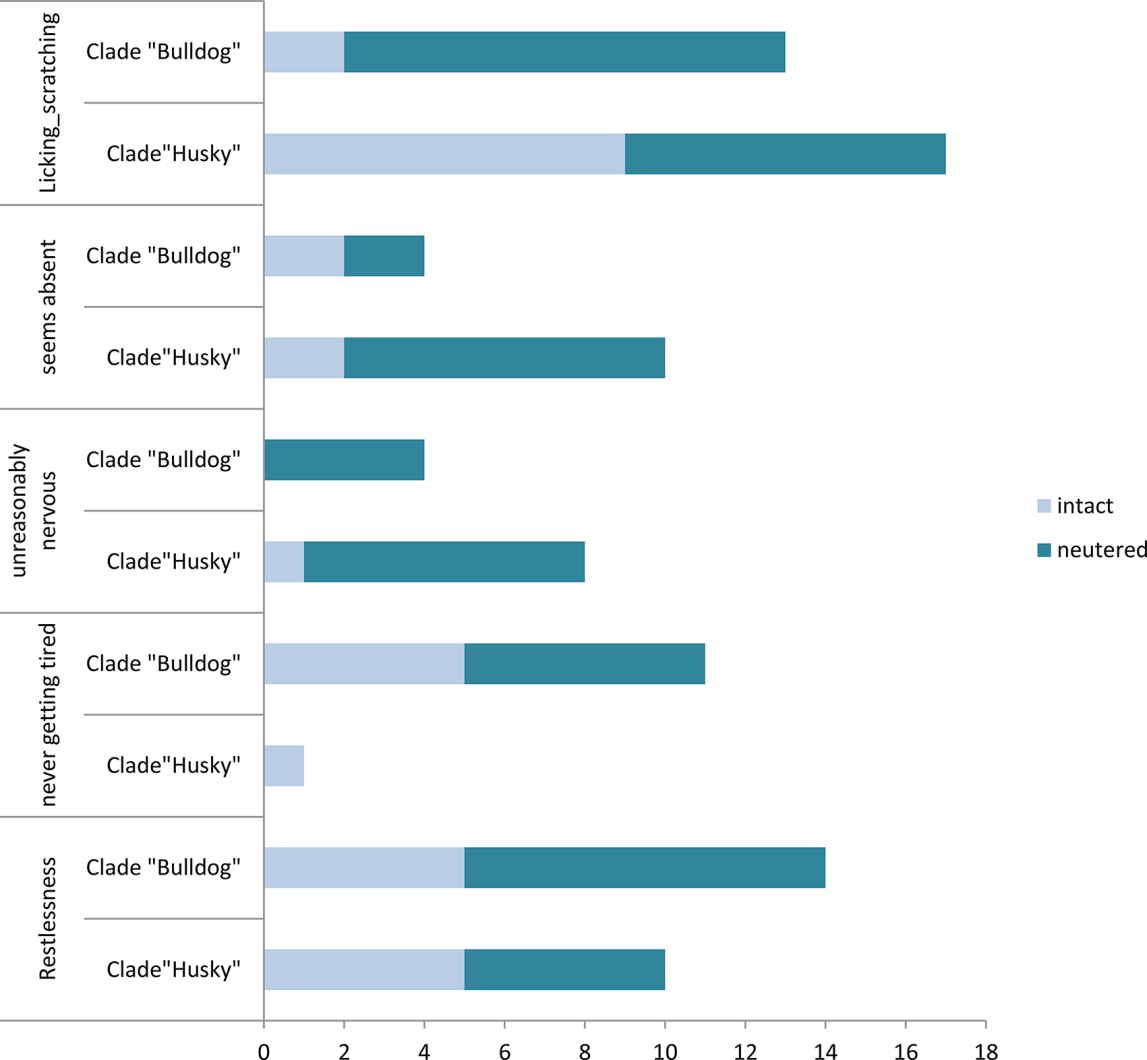
Nervous Behaviour: Comparison of neutered (*n* = 31) and intact males (*n* = 37) of the clade “Huskies” with the neutered (*n* = 30) and intact males (*n* = 38) of the “Bulldog” clade regarding nervous behaviour depending on their breed and neutering status

As shown in Fig. 3, excessive licking and scratching can be noted for more “Huskies” and for more neutered than intact dogs (multinomial regression analysis: *p* = 0.05, Cramer’s V = 0.21 for both variables; for neuter status: *p* = 0.02). The exaggerated licking and scratching are primarily shown by the neutered representatives of the “Bulldog” clade. More “Bulldogs” show restless behaviour, whereas more “Huskies” seem to be absent and show nervous behaviour. In seeming absent there were significant differences for both variables (multinomial regression analysis: *p* = 0.02; Cramer’s V = 0.23) as well as significant differences between castrated and intact dogs (multinomial regression analysis: *p* = 0.03; OD = 2.8). There were also significant differences regarding the dog getting tired (multinomial regression analysis: *p* = 0.005). Here it was mainly the “Bulldogs” for which the significance was decisive (multinomial regression analysis: *p* = 0.001). The males also differed in their nervous behaviour (multinomial regression analysis: *p* < 0.001; V = 0.33) and the neutered ones in particular seemed more nervously (multinomial regression analysis: *p* < 0.001).

### Budapest questionnaire

**Fig. 4:**
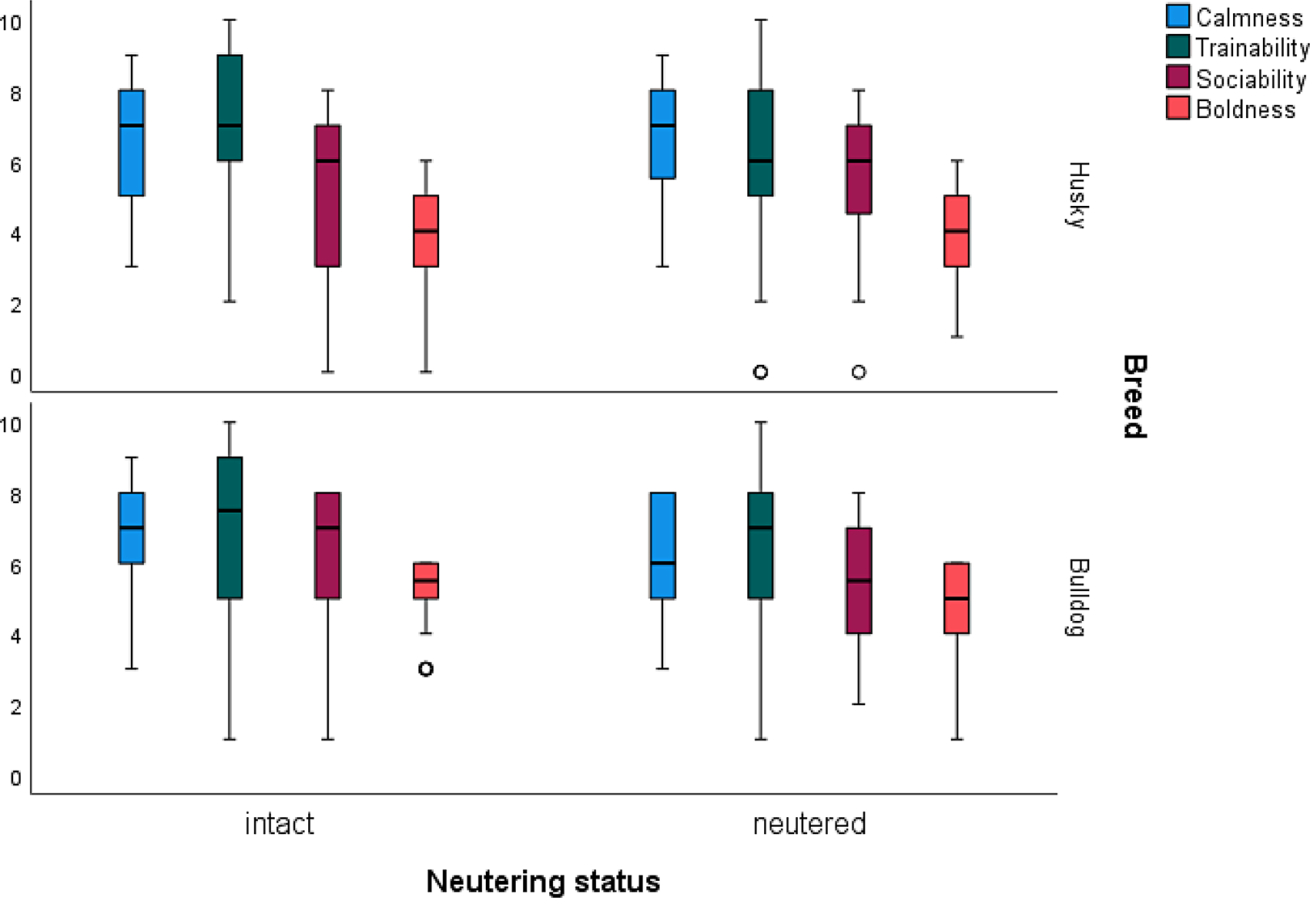
Results from the Budapest questionnaire of neutered (*n* = 31) and intact males (*n* = 37) of the clade “Huskies” and the neutered (*n* = 30) and intact males (*n* = 38) of the “Bulldogs” clade

The results of the Budapest questionnaires (Fig. 4) showed that the “Bulldog” clade scored significantly higher overall for extraversion (ordinal regression analysis, *p* < 0.001; OD = 3.21). Within the “Bulldog” clade, the intact males were more extroverted than the neutered males. Concerning calmness, a small but not significant difference between neutered and intact Bulldogs was found, in that the neutered ones are less calm than the intact ones. A similar result emerges for trainability within the “Husky” clade, whereas the intact “Bulldogs” differed somewhat from the intact ones in that their trainability values were slightly higher than those of the neutered dogs. In sociability, there was a difference between the neutered and intact Bulldogs, with the intact Bulldogs appearing more sociable than the neutered dogs of the same breed (Fig. 4).

The statistical results of the data analyses are summarised in Table 3. The following results of the multinomial and ordinal regression analyses and trends are relevant:

**Table 3:** Statistical results of multinomial and ordinal regression analyses of the categories with significant p-values and trends. All effect sizes (Cramer’S V and Odd’s ratio (OD)) are marked white/grey depending on their bandwidth (small, medium, large and very large)

Variable	Final p-value (Combined effects)	Neuter status *p*-value	Breed *p*-value	Cramer’s V (Combined effects)	OD (Neuter status)	OD (Breed)
**Multinomial regression analysis**						
Aggression	0.06	0.06	n.s.	0.2	0.32	2.46
Aggression_dogs	0.03	0.08	0.04	0.23	0.52	2.2
Aggression_humans	0.006	0.002	n.s.	0.28	0.08	0.61
Aggr_humans_ household	0.03	n.s.	0.02	0.23	0.26	1.2 E-8
Destroying objects	0.05	0.1	0.06	0.21	0.46	0.3
Licking_scratching	0.05	0.02	n.s.	0.21	0.38	1.4
Nervous	< 0.001	< 0.001	n.s.	0.33	0.06	2.21
Never getting tired	0.005	n.s.	0.001	0.28	0.76	0.08
Panic	n.s	0.05	n.s.	0.17	0.15	0.98
Pulling on leash	0.08	0.07	n.s.	0.2	2.08	1.73
Seems absent	0.02	0.03	0.09	0.23	0.28	2.8
Stress_dogs	0.02	0.004	n.s.	0.25	0.27	0.79
Stress_noises	0.02	n.s.	0.006	0.24	0.8	5.11
Stress_uncertainty	< 0.001	< 0.001	n.s	*0.42*	0.09	1.35
Thyroid	0.1	n.s.	0.04	0.18	0.6	6.5
**Ordinal regression analysis**						
Extraversion	< 0.001	n.s.	< 0.001	0.32	0.85	3.21

## Discussion

Many of our results are consistent with other study findings. With regard to aggression towards humans, the results of the questionnaire showed a significance in that castrated males behaved aggressively more frequently than intact males. Kaufmann et al. [7] also indicated in their study that castrated male dogs were more likely to be aggressive towards humans. Lorenz et al. [8, 9] found something similar in female dogs.

Farhoody and Zink [34] investigated the effects of neutering and found a negative impact on dog behaviour. Neutered female dogs were more aggressive and excitable than their intact conspecifics. The questionnaire study by Hsu and Sun [49] based on C-BARQ also showed that neutered male and female dogs are more aggressive towards their owners than intact dogs. Strodtbeck and Gansloßer [36] provide a possible explanation for these findings, namely the cortisol-testosterone balance, in which the two hormones compete with each other [25].

Nevertheless, our results also contrast with the results of other studies in which either no effect [14–16] or even a positive effect of castration on dog behaviour was recorded [17, 18].

Our results also showed that there are breed-related differences in aggressive behaviour. In this case, it was the “Huskies” that showed more aggressive behaviour towards other dogs and towards humans of the same household. Huskies are often described as bold. Essential character traits that often belong to Huskies are their social compatibility, fearlessness and curiosity [42].

Despite these character descriptions, the “Huskies” in our study were noted as more aggressive towards their conspecifics. Similar results were obtained in a Polish study on undesirable behaviour in ancient dog breeds (such as Akita, Alaskan Malamute, Basenji, Samoyed and Siberian Husky). According to this study, undesirable behaviours, such as aggression towards humans and other dogs/animals, separation anxiety, excessive vocalization, oral and locomotion behaviours occur mainly in Akitas, Siberian Huskies and Samoyeds. And it is mainly males that are more affected. Since our study also included primarily Huskies, our results support the data of Wójcik and Powierza [50].

Studies on behavioural problems in Huskies are scarce, they represent only 0.43% of the populations studied and yet show the same behavioural problems as the most popular dog breeds [50].

Parker et al. [51] describe Akitas, Malamutes and Huskies as independent, intelligent, social, active characters with a high urge to move. In addition, they are very territorial and have a strong hunting behaviour. Accordingly, these breeds certainly need to be trained differently. Dogs tend to exhibit undesirable behaviour when their biological and behavioural needs are not met [52]. Likewise, aggression towards other dogs may also be related to leash handling. Especially these agile dogs like Huskies and Akitas seem not to be very secure on a short leash and in some cases their aggressive behaviour could also be an expression of their insecurity or, in case of poor bond with the dog owner, their defence of owner [50, 53].

Furthermore, the individual personality and handling by the owner have a significant effect on the dog’s behaviour and can promote aggression under certain circumstances [54]. According to an American study [55] more neutered than intact dogs tend to behave aggressively and to attack or bite other dogs.

Some other studies also point to a possible correlation between castration and aggressive behaviour [56, 57].

Nevertheless, the connection between sex hormones and aggressive behaviour seems to be even more complex than previously assumed. Hence, it is extremely important to distinguish between the different forms of aggression as there are differences in the risk factors and the prevention or treatment of bite injuries. The prognosis can also be different for the various categories of aggression [58, 59].

Given the complexity of domestic dog aggressiveness, De Keuster and Jung’s diagnosis of “intra-specific affective aggression” may be more applicable [60].This form of aggression is primarily manifested as protective aggression [21, 58] or alsoknown as “social aggression,“, which is linked to a high degree of sympathetic arousal and can be either aggressive (self-confident) or defensive (fearful). The aim of affective aggression is to put more distance between the subject and a danger or annoyance [61].

It is formerly known as “dominance or status-related aggression” and is characterised by “dominant” and rather offensive aggressive behaviour [21]. Van der Borg et al. [62] describe the term dominance as “dominance hierarchies” and tend to assume “dominance relationships” among dogs. These primarily include status assesments and specific body postures such as dominant and submissive behaviour. For example, formal dominance tends to include submissive elements rather than aggression [63].

Affective aggression is directed against a family member (human and/or dog) and depends on many factors, as already described above: On the relationship to the current and possibly to the first owner and in particular on the type of education (punishment, reward, consequence etc.) [64].

These types of dogs often have a history of early illness and of excitability and anxiety as puppies. Even in adulthood, they are still more excitable and anxious [63].

The neurotransmitters serotonin, dopamine and noradrenaline are primarily involved in affective aggression [65–67]. With regard to defensive aggression it can be said that the stress hormone systems, especially noradrenaline, is also involved in this type of aggressive behaviour without a connection to the sex hormone testosterone. Similarly, in male wolves, territorial aggression is not controlled by this sex hormone [68]. Castration also has no positive effects on defensive behaviour of males with regard to puppies. Rather, the removal of the testicles amplifies the effect of testosterone produced in the adrenal glands on such behaviours, since it works more effectively in small amounts together with prolactin. Similarly with regard to social bonding, jealousy or partner-protective behaviour is social, not sexual, and is influenced by the hormone vasopressin [69, 70].

There is also serotonin-dependent aggressive behaviour. If the serotonin level is too low, aggressive behaviour can be encouraged. The assumption is a connection between sex hormones and serotonin [66, 71, 72].

Hart and Hart [73] identify the Husky belonging to the cluster of low reactivity, high aggression and low trainability. The Bulldogs were assigned to very low reactivity, very low aggression and low trainability. They also suggest that breeds being high on one form of aggression are also prone to other forms of aggression.

Our data support the hypothesis (H1: Neutered males are (breed-dependently) more often aggressive than intact males) in particular, that human-directed aggression occurs in more neutered than intact dogs.

The second hypothesis (Neutered males show (breed-dependently) more stress-indicating behaviour than intact males) can also be supported by our data.

The neutered males were overall more stressed and more insecure in their behaviour than the intact dogs. The results were significant for the overall analysis and even more significant depending on the castration status. A significant correlation with a high odds ratio for stress in relation to noises could be found in more “Huskies” than “Bulldogs”.

This increased stress and insecurity of neutered dogs again give an indication of the behavioural-biological consequences of gonadectomy, which was also identified by Zink et al. [74] in neutered Vizslas.

Here again there could be a connection with cortisol. The pulsatile characteristics of cortisol can cause increased stress in animals. However, Seale et al. [75] were able to demonstrate that this hormonal imbalance can be counteracted by externally supplied sex hormones. The basal and stress-induced corticosterone levels decreased again in rats after hormone replacement.

Several studies have shown that neutered dogs are more excited and fearful than intact ones [7–10, 16, 34]. A review of medical files and online surveys found that neutered dogs are more likely to have separation anxiety and fear of storms than intact dogs [76]. Additionally, in an experimental study of 38 dogs divided into three groups (1st. intact dogs, 2nd. neutered at seven weeks and 3rd. neutered at seven months), all neutered dogs were generally more active and the males neutered at seven weeks were more excitable than the intact ones [77]. Again, Bennett and Rohlf [78] discovered that neutered males and females were more anxious, nervous and showed more destructiveness. Storengen and Lingaas [79] came to a similar conclusion. In their online study the neutered were more fearful of noises than the intact dogs. However, the authors hypothesize that this could be due to the fact that in their study it was mainly neutered males that were affected by increased anxiety and that they are often neutered because of such or general behavioural problems [79].

The questionnaire and video analyses studies by Kaufmann et al. [7] and Lorenz et al. [8, 9] came to similar conclusions. Again, it was the neutered male and female dogs that were more stressed, anxious and panicky. In the dog breed study by Kolkmeyer et al. [10] with a similar study design, in which four different breed clades (“Terriers”, “Retrievers”, “Hunting dogs”, “Shepherds”) were investigated, again the castrated males showed increased panic behaviour regardless of their breed.

There was also a trend for increased panting in the neutered males in our study.

When under extreme (heat) stress, the path of airflow, which involves mouth and nose inhalation, is switched on to increase the ventilatory rates [80]. It seems that both the rate of salivation and lingual blood flow are regulated by thermoregulatory systems [81, 82].

Panting can also be an indication of anxiety in dogs [83].Our results indicate that panting occurred in more neutered than intact dogs. On the basis of the above mentioned data it could be explained by the castration-related loss of sex hormones and the resulting increase in stress or anxiety [84]. Increased anxiety accompanied by increased panting also occurred in the study by Tiira et al. [61]. Another assumption could be, that this intense panting could be due to an altered thermal balance in the neuters, but there is still a lack of research data.

Our data did not show any significant results in favour of the positive effects of castration. The loss of hormones results in an imbalance and can lead to behavioural problems such as stress/insecurity and aggression. No significant differences were found between the dog breeds except for extraversion. As already described above, not all personality traits and behaviours are genetically embodied in such a way that they only occur in a breed-specific manner.

A dog’s personality is difficult to define directly, but can be described by various character traits such as those mentioned above [82]. Male dogs are often neutered with the aim of eliminating undesirable behaviours such as mounting, straying, aggressiveness and urine marking. However, the extent to which a behaviour pattern changes as a result of neutering is not clearly defined [85] and our data underline the risk of neutering and the side effects (like increased fear, aggression or stress-related behaviour) that can occur. In no way does gonadectomy replace adequate training or bonding and proper socialisation of the dog.

The effects of neutering seem to depend more on the dog’s personality than just the breed and do not affect every behaviour pattern, as not all behaviours depend on sex hormones.

Finally, there are some important limitations in this study that need to be considered. Due to the small sample size, no general conclusions can be drawn. In addition to the sample size, a more equal distribution of dog breeds would have been desirable. Consequently, since certain dog breeds such as “Husky” or “Bulldog” are more popular than others from the two clades, the corresponding distribution occurred. Since no pedigree of the participating dogs was requested, the reliability of the results should also be considered.

### Electronic supplementary material

Below is the link to the electronic supplementary material.

Supplementary Material 1

Supplementary Material 2

## Data Availability

The raw data presented in this study is also available on request from the corresponding author.

## Declarations

**Statement of experimental control**.

The study does not include an experimental design, as only questionnaires about the dogs were completed.

**Declaration of conformity with the relevant guidelines**.

The online survey method used is in accordance with the relevant guidelines and regulations.
